# Influence of Menstrual Cycle or Hormonal Contraceptive Phase on Physiological Variables Monitored During Treadmill Testing

**DOI:** 10.3389/fphys.2021.761760

**Published:** 2021-12-16

**Authors:** Ritva S. Taipale-Mikkonen, Anna Raitanen, Anthony C. Hackney, Guro Strøm Solli, Maarit Valtonen, Heikki Peltonen, Kerry McGawley, Heikki Kyröläinen, Johanna K. Ihalainen

**Affiliations:** ^1^Sports Technology Unit, Faculty of Sport and Health Sciences, University of Jyväskylä, Vuokatti, Finland; ^2^Faculty of Sport and Health Sciences, University of Jyväskylä, Jyväskylä, Finland; ^3^Department of Exercise and Sport Science, The University of North Carolina at Chapel Hill, Chapel Hill, NC, United States; ^4^Department of Nutrition, The University of North Carolina at Chapel Hill, Chapel Hill, NC, United States; ^5^Department of Sports Science and Physical Education, Nord University, Bodø, Norway; ^6^School of Sport Sciences, UiT The Arctic University of Norway, Tromsø, Norway; ^7^Research Institute for Olympic Sports (KIHU), Jyväskylä, Finland; ^8^Swedish Winter Sports Research Centre, Department of Health Sciences, Mid Sweden University, Östersund, Sweden

**Keywords:** endurance testing, menstrual cycle, hormonal contraceptives, aerobic testing, female physiology

## Abstract

**Purpose:** To examine the influence of menstrual cycle (MC) and hormonal contraceptive (HC) cycle phases on physiological variables monitored during incremental treadmill testing in physically active women (eumenorrheic, EUM = 16 and monophasic HC-users, CHC = 12).

**Methods:** Four running tests to exhaustion were performed at bleeding, mid follicular (mid FOL)/active 1, ovulation/active 2, and mid luteal (mid LUT)/inactive. HC and MC phases were confirmed from serum hormones. Heart rate (HR), blood lactate (Bla), and $\dot{\text{V}}$O_2_ were monitored, while aerobic (AerT) and anaerobic (AnaT) thresholds were determined. $\dot{\text{V}}$O_2peak_, maximal running speed (RUN_peak_), and total running time (RUN_total_) were recorded.

**Results:** No significant changes were observed in $\dot{\text{V}}$O_2_ or Bla at AerT or AnaT across phases in either group. At maximal effort, absolute and relative $\dot{\text{V}}$O_2peak_, RUN_peak_, and RUN_total_ remained stable across phases in both groups. No significant fluctuations in HR_max_ were observed across phases, but HR at both AerT and AnaT tended to be lower in EUM than in CHC across phases.

**Conclusion:** Hormonal fluctuations over the MC and HC do not systematically influence physiological variables monitored during incremental treadmill testing. Between group differences in HR at AerT and AnaT underline why HR-based training should be prescribed individually, while recording of MC or HC use when testing should be encouraged as phase may explain minor, but possibly meaningful, changes in, e.g., Bla concentrations or differences in HR response.

## Introduction

Incremental aerobic treadmill testing is an essential tool for determining cardiorespiratory fitness and/or monitoring training adaptations. Accordingly, test results are commonly used to evaluate, prescribe, and adjust training for athletes. Among commonly investigated physiological variables are peak oxygen uptake ($\dot{\text{V}}$O_2peak_) as well as heart rate (HR), blood lactate concentration (Bla), respiratory exchange ratio (RER), ventilation rate (VE), peak running speed (RUN_peak_), total running time (RUN_total_), and rating of perceived exertion (RPE). Aerobic (AerT) and anaerobic (AnaT) thresholds are often determined (Aunola and Rusko, 1984, 1986) due to their importance in exercise prescription. Several factors can affect physiological variables and testing outcomes, such as training status, psychological state, glycogen stores, level of recovery, etc. Moreover, some level of biological variation is expected between repeated tests that may not be directly attributed to training or detraining (Bagger et al., 2003). For female athletes, it can be hypothesized that the fluctuation of endogenous sex hormones (e.g., estrogen, E2, and progesterone, P4) associated with the menstrual cycle (MC) or hormonal contraceptive (HC) use (i.e., exogenous hormones) might influence physiological variables and/or performance due to their non-reproductive actions on the cardiovascular system and substrate metabolism as described in the following paragraph.

A eumenorrheic MC is typically divided into two basic phases: the follicular phase (FOL), which is characterized by low concentrations of E2 and P4, and the luteal phase (LUT), which is characterized by high concentrations of E2 and P4 (Davis and Hackney, 2017). Ovulation commonly occurs between FOL and LUT and is marked by a surge in luteinizing hormone (LH) and E2, as well as a smaller surge in follicle-stimulating hormone (FSH). Monophasic HCs suppress hypothalamic-pituitary-ovarian (HPO) axis function (Elliott-Sale et al., 2013). In the skeletal muscle and cardiac tissues, E2 may cause vasodilation (Mendelsohn and Karas, 1999) that increases in parallel to E2 concentrations at rest (Kawano et al., 1996). During exercise between 40-100% maximal oxygen consumption (VO_2max_), a tendency for increased plasma volume, and a concomitant increase in pulmonary diffusion capacity associated with an increase E2 may also be observed (Smith et al., 2015). Progesterone appears to attenuate the effects of E2 (Mendelsohn, 2002). Higher levels of P4 (i.e., mid LUT) in eumenorrheic women have been linked to increased HR, VE, and core temperature at rest, although MC phase (mid FOL versus mid LUT) does not appear to affect VE (MacNutt et al., 2012), HR, O_2_ uptake, or CO_2_ output during either submaximal or strenuous exercise (Jurkowski et al., 1981). Metabolically, E2 has been shown to spare glycogen and increase fat oxidation (Hackney, 1999) (reflected as lower Bla and RER values), although conditions in which P4 is also high (i.e., mid LUT) may mitigate these effects (D’Eon et al., 2002). Monophasic HCs do not appear to influence VO_2max_ or RUN_total_ (Bryner, 1996), however, they may induce a glycogen-sparing effect (Bonen et al., 1995). In rowers, power output, HR, $\dot{\text{V}}$O_2_, CO_2_ production, VE, mean RER, and ventilatory equivalents of O_2_ and CO_2_ did not differ between active and inactive HC phases (Vaiksaar et al., 2011); however, the active phase significantly increased VE, breathing frequency, and VE for O_2_ and CO_2_ in endurance trained women (Barba-Moreno et al., 2019). In theory, these observations regarding the effects of endogenous and exogenous reproductive hormones on skeletal muscle and cardiac tissues, metabolism, and cardiovascular responses suggest that MC phase and/or HC use could affect physiological variables monitored during incremental exercise testing. Indeed, the flux of hormones during the MC has been reported to affect cardiorespiratory function, training responses and adaptations, recovery from exercise (Ihalainen et al., 2019), and performance (Davies et al., 1991). In the context of exercise testing, Smekal et al. (2007) reported no effect of the MC on $\dot{\text{V}}$O_2peak_, while Lebrun et al. (1995) reported a higher absolute $\dot{\text{V}}$O_2peak_ in the early FOL than mid LUT, although these differences may be attributed, in part, to methodological approaches.

At present, the quality of evidence regarding the variation in physiological variables monitored during incremental treadmill testing and/or performance outcomes across the MC is relatively low (McNulty et al., 2020). This topic requires more research with improved methodological quality, including the confirmation of MC phases and reporting HC types and dosages used by participants. Similarly, the majority of research focusing on HC use is of low quality and investigates and compares the differences between HC use and non-use (Elliott-Sale et al., 2020a), rather than examining potential changes in physiological variables monitored during testing and/or performance over the HC. Although recent meta-analyses found trivial changes in performance across the MC (McNulty et al., 2020) and minor effects of HC use on performance, in general (Elliott-Sale et al., 2020a), it is essential to consider the large between-study variance as well (Elliott-Sale et al., 2020a; McNulty et al., 2020). Considering the contradictory findings described above as well as the importance of the physiological variables evaluated during incremental testing, in the context of sports training and monitoring, the purpose of this study was to investigate the influence of MC phase, and comparable time-points in the HC-cycle, on physiological variables monitored during incremental treadmill testing in physically active eumenorrheic and monophasic HC-using women. Based on previous observations, we hypothesized that small but possibly meaningful fluctuations in physiological parameters, such as decreased RER and lactate in the luteal phase, might be observed in eumenorrheic women. We also hypothesized that the measured physiological variables would remain relatively stable in women using monophasic HCs.

## Materials and Methods

### Participants

Healthy women, age 18–40 years, were recruited by advertisements in the local newspaper and *via* social media. Before inclusion in the study, each prospective participant was asked to complete a health questionnaire and a Low Energy Availability in Females Questionnaire (LEAF-Q) (Melin et al., 2014). Inclusion criteria required that participants be physically active (strength training 3 times⋅week^–1^ and endurance training 3 times⋅week^–1^) with a BMI of 18–25 kg⋅m^–2^ and a LEAF-Q score <8. Participants were excluded if they were pregnant or lactating, if they had conditions affecting ovarian function, amenorrhea, endocrine disorders, or chronic diseases, or if they were taking medication that may have affected exercise responses. Participants received detailed information about the study design, measurements, and procedures before signing an informed consent document. Participants were aware that they could withdraw from the intervention at any time. The data presented are part of a larger endogenous and exogenous hormones and performance in women (MEndEx) study, which was approved by the Ethical Committee at the University of Jyväskylä, Finland on October 22, 2018.

A total of 33 women were enrolled in the study. Five participants dropped out prior to the completion of the study due to personal reasons or schedule conflicts. Data were ultimately analyzed and are presented for *n* = 28. Descriptive data (gathered at bleeding, see study design), including participant characteristics, are presented in Table 1. Participants included women who had an MC classified as eumenorrheic and had not used a HC for at least one year (EUM = 16) and women who had used a monophasic combined synthetic estrogen and progestin HC for at least one year (CHC = 12). The monophasic contraceptives used by participants are listed in Table 2.

**TABLE 1:** Participant information for the eumenorrheic (EUM) women and the monophasic hormonal contraceptive users (CHC).

	EUM*n* = 16	CHC*n* = 12
Age (years)	26 ± 4	23 ± 2
Body mass (kg)	67.9 ± 7.0	62.8 ± 5.1
Height (cm)	167.1 ± 5.6	170.0 ± 5.6
Body fat (%)	21.8 ± 6.6	19.2 ± 3.2
LEAF-Q (points)	3.8 ± 2.7	5.1 ± 1.8
Length of menstrual cycle (days)	28.3 ± 2.3	28

**TABLE 2:** Brand and dosage of hormonal contraceptives used by participants in the monophasic hormonal contraceptive user group (CHC).

*N*	Brand name	Dose
5	Yaz, Tasminetta, Stefaminelle	0.02 mg ethinyl estradiol/3 mg drospirenone
3	Yasmin	0.03 mg ethinyl estradiol/3 mg drospirenone
1	Zoley	2.5 mg nomegestrol/1.5 mg estradiol
2	Vreya	0.035 mg ethinylestradiolum/2 mg cyproteron acetate
1	Nuvaring	0.120 mg etonogestrel/0.015 mg ethinyl estradiol

### Study Design

**FIGURE 1:**
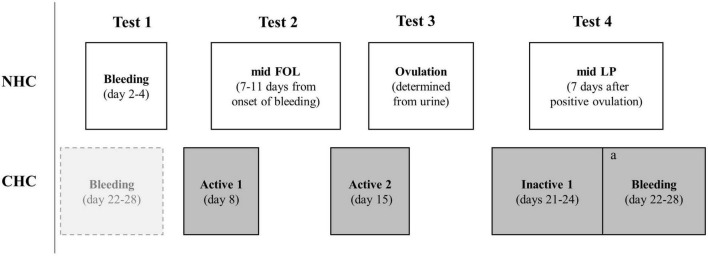
Overview of the timing of incremental treadmill testing in the eumenorrheic (EUM) participants and the monophasic hormonal contraceptive users (CHC). a = 3–6 days of habitual physical activity or active rest separated inactive 1 and bleeding tests.

Four experimental testing sessions were completed by each participant over an individual MC or HC-cycle with the timing of testing illustrated in Figure 1. The phase of the MC or HC-cycle in which testing commenced was randomized. Procedures were performed according to current recommendations for best practice (Elliott-Sale et al., 2020b). Ovulation was identified using daily urine tests completed by the participant at home, starting mid FOL, to identify the LH surge (Dipro, LH Ovulation Strip, Aidian Oy, Finland). Ovulation was detected in all EUM participants and MC phases were retrospectively confirmed by analysis of serum hormones. Tests were scheduled 1–2 days after ovulation was detected. Cycle length (Table 1) was within clinical norms for all participants (Hampson, 2020). Menstrual bleeding and withdrawal bleeding are simply referred to as “bleeding” throughout the manuscript. For practical purposes, MC and HC-cycles were compared at bleeding, mid FOL/active 1, ovulation/active 2, and mid LUT/the inactive phase.

### Incremental Treadmill Running Testing

A treadmill test was performed to assess physiological variables associated with aerobic capacity using a standard incremental protocol (Mikkola et al., 2007). A resting fingertip blood sample was taken for the analysis of resting Bla (EKF diagnostic, C-line system, Biosen, Germany). Treadmill incline remained constant at 0.5° for the entire test. Treadmill velocity was 6 km⋅h^–1^ for the first 3-min stage of the test and was increased by 1 km⋅h^–1^ every third min until volitional exhaustion. Fingertip blood samples, for the subsequent analysis of submaximal Bla, were taken between each stage when the treadmill was briefly stopped for 30-s. HR was recorded continuously using a HR monitor (Polar 800, *Polar Electro*, Kempele, Finland). Mean HR and $\dot{\text{V}}$O_2_ values from the last 30-s of each stage were used for analysis. The $\dot{\text{V}}$O_2_ was measured breath-by-breath using a portable gas analyzer (calibrated according to manufacturer instructions, Oxycon Mobile^®^, Jaeger, Hoechberg, Germany) and $\dot{\text{V}}$O_2peak_ was defined as the highest average 30-s $\dot{\text{V}}$O_2_ value. The AerT and AnaT were determined from Bla and gas exchange variables according to Aunola and Rusko (1986) a method previously shown to be reproducible and reliable (Aunola and Rusko, 1984). The RUN_peak_ was defined as the highest treadmill speed maintained for >30 s and RUN_total_ was measured from the start of the first stage until the participant reached exhaustion, when the treadmill was subsequently stopped. HR_max_ was recorded as the highest 5-s HR value. Testing was completed at the same time of day ±1 h to avoid the possible confounding effects of e.g., circadian rhythms. Furthermore, participants were instructed to refrain from strenuous exercise for the 24 h prior to testing.

### Nutrition

Participants were instructed to maintain their typical diet throughout the study and to continue eating as they normally would, *ad libitum*. A 3-day food diary including the day before, day of, and day after aerobic testing was collected for each phase. Analysis of food diaries using software (Fineli, National Institute for Health and Welfare, Helsinki, Finland) indicated no significant differences in total energy or macronutrient intake between tests (see Ihalainen et al., 2021). Prior to testing, participants were instructed to avoid caffeine and to eat a typical light meal or snack roughly 3 h before the test. Strength testing, including maximal voluntary contractions and a loading of explosive leg press (2 × 10 at 60%1RM load with 2 min recovery between sets) was completed prior to endurance testing and was followed by 15-min rest. While strength testing may induce some fatigue, participants were accustomed to physical exertion were adequately recovered upon commencement of endurance testing (lactate at pre was 1.37 ± 0.46 mmol⋅L^–1^ in EUM and 1.43 ± 0.41 mmol⋅L^–1^ in CHC). Participants were offered an energy bar (Isostar High Energy Sport Bar, multi fruit) and 1.5 dl of water prior to aerobic testing. If the participant chose to eat the energy bar (or part of the energy bar) prior to aerobic testing, this was repeated during all trials.

### Body Composition

Body composition was assessed in a 12-h fasted state in the morning between 07:00 and 09:00 prior to aerobic testing using a multi-frequency bioelectrical impedance analyzer (InBody 720; Biospace, Seoul, South Korea) with participants wearing only underwear. To reduce the potential for influencing eating behaviors, participants were not given feedback regarding their body composition results until the study was completed.

### Blood Samples

Blood samples were collected in a 12-h fasted state in the morning between 07:00 and 09:00 prior to testing. Samples were taken from the antecubital vein into serum tubes (9 ml Venosafe Gel + Clot activator tubes, Terumo Medical Co., Belgium and 6 ml Venosafe EDTA Tubes, Terumo Medical Co., Belgium). Each participant’s basic blood count (analyzed from blood samples in the EDTA tubes by Sysmex KX-21N, Kobe, Japan) was evaluated for indication of acute illness/infection. The samples in the Gel + Clot activator tubes were centrifuged for 10 min at 2000 × *g* and a refrigerated temperature of +4°C (Heraeus Megafuge 1.0 R, Thermo Scientific, Karsruhe, Germany). The serum was separated and immediately frozen at −80°C for later analysis of E2, P4, LH, and FSH. Hormonal analyses were performed using chemical luminescence techniques (Immulite 2000) with an assay sensitivity of 55.0 pmol⋅ L^–1^ for E2, 0.3 ng⋅ml^–1^ for P4, 0.05 mIU⋅L^–1^ for LH, and 0.10 IU⋅L^–1^ for FSH. Inter-assay coefficients of variation were 6.7% for E2, 9.7% for P4, 4.8% for LH, and 3.4% FSH.

### Statistical Analysis

Bleeding, mid FOL and active 1, ovulation and active 2, and mid LUT and the inactive phase of the HC cycle were “matched” for the sake of reporting and analysis. Mean values and standard deviations (±SD) were calculated using standard methods. Statistical analyses were completed using IBM SPSS Statistics 26.0 (IBM Corporation, IBM SPSS Statistics for Windows, Armonk, New York, United States). Data was normally distributed. A factorial mixed design ANOVA, including 1 between-subject factor (groups: EUM and CHC) × 1 within-subject factor (phase: bleeding, mid FOL/active 1, ovulation/active 2, and mid LUT/the inactive phase) was performed. In the presence of a main effect for phase or group, simple main effects (the effect of phase on EUM or CHC groups alone or the effect of group over phase) pooled for error term *via* MANOVA were completed to determine if the main effect can be justifiably interpreted. In the presence of an interaction, 2 × 2 mixed design ANOVAs were performed to identify where the interaction resides along the independent variable (phase). Mauchly’s test was used to test the assumption of sphericity. Where this assumption was violated, Greenhouse–Geisser adjustments were applied. Statistical significance was set at *p* ≤ 0.05. Due to the sample size being <20, effect sizes were estimated using Hedges’ *g* where values of <0.25, 0.25–0.5, 0.5–1.0, and >1.0 were interpreted as trivial, small, medium, and large, respectively.

## Results

### Female Reproductive Hormones During the Menstrual and Hormonal Contraceptive Cycles

Serum hormone concentrations measured for EUM and CHC in the present study (Table 3) are reflective of normal eumenorrheic MC and HC-cycles, respectively.

**TABLE 3:** Serum concentrations of estradiol (E2), progesterone (P4), luteinizing hormone (LH), and follicle-stimulating hormone (FSH) across the four cycle phases for the non-hormonal contraceptive (EUM) and hormonal contraceptive (CHC) groups.

	EUM				CHC				ANOVA		
	Bleeding	Mid FOL	Ovulation	Mid LUT	Bleeding	Active 1	Active 2	Inactive	Phase	Group	Phase × group
E2 (pmol⋅L^–1^)	285 ± 139	537 ± 381	689 ± 479 *	669 ± 233 *	300 ± 270	190 ± 138	217 ± 234 *	189 ± 108 *	*F* = 2.575 *p* = 0.084	***F* = 12.340** ***p* = 0.002**	***F* = 3.519** ***p* = 0.035**
P4 (nmol⋅L^–1^)	1.94 ± 1.64	1.02 ± 0.43	4.08 ± 2.58 *	14.77 ± 8.4 *	1.05 ± 0.52	1.00 ± 0.48	1.14 ± 0.97 *	1.18 ± 1.01 *	***F* = 16.986** ***p* < 0.001**	***F* = 21.392** ***p* < 0.001**	***F* = 15.572** ***p* < 0.001**
LH (mIU⋅L^–1^)	5.77 ± 3.32	6.85 ± 2.77 *	14.16 ± 13.04 *	4.32 ± 2.80	3.75 ± 2.87	2.97 ± 3.17 *	1.79 ± 2.08 *	2.54 ± 2.42	*F* = 3.386 *p* = 0.069	***F* = 7.813** ***p* = 0.011**	***F* = 5.000** ***p* = 0.028**
FSH (IU⋅L^–1^)	5.43 ± 2.48	6.62 ± 2.73 *	6.82 ± 2.99 *	2.81 ± 1.23	4.36 ± 2.24	2.34 ± 1.81 *	1.82 ± 1.61 *	2.86 ± 2.40	***F* = 6.048** ***p* = 0.001**	***F* = 10.458** ***p* = 0.004**	***F* = 7.523** ***p* < 0.001**

Values are presented as mean ± SD and significant ANOVA findings are denoted in bold. Simple main effects for phase were observed for E2, P4, LH, and FSH in EUM (F = 7.62, p < 0.001; F = 41.56, p < 0.001; F = 10.42, p < 0.001; and F = 13.75, p < 0.001) but not in CHC (F = 0.11, p = 0.956; F = 0.02, p = 0.995; F = 0.19, p = 0.903; and F = 2.31, p = 0.085). Simple main effects for group at each phase are denoted by “*” in the table (p < 0.05).

### Incremental Treadmill Running Testing

Body mass, physiological and aerobic performance test variables are presented in Table 4 for EUM and CHC. No within-group or between-group differences were observed in body mass.

**TABLE 4:** Heart rate, blood lactate, and $\dot{\text{V}}$O_2_ at aerobic and anaerobic thresholds as well as maximal effort across the four cycle phases for the eumenorrheic (EUM) and the hormonal contraceptive (CHC) participants.

	EUM				CHC				ANOVA		
	Bleeding	Mid FOL	Ovulation	Mid LUT	Bleeding	Active 1	Active 2	Inactive	Phase	Group	Phase × group
Body mass (kg)	67.1 ± 7.8	66.4 ± 7.9	66.7 ± 8.2	66.6 ± 7.6	63.5 ± 6.0	63.1 ± 6.1	63.3 ± 8.2	63.5 ± 5.8	*F* = 1.714 *p* = 0.174	*F* = 3.869 *p* = 0.063	*F* = 0.983 *p* = 0.392
Blood glucose (mmol⋅L^–1^)	5.0 ± 0.3	5.1 ± 0.4	5.0 ± 0.4	4.9 ± 0.4	4.8 ± 0.4	4.7 ± 0.4	4.9 ± 0.4	4.9 ± 0.3	*F* = 0.164 *p* = 0.921	*F* = 1.081 *p* = 0.309	*F* = 2.073 *p* = 0.112
**Aerobic threshold**											
Heart rate (bpm)	154 ± 14 *	155 ± 13 *	156 ± 12 *	156 ± 11 +	166 ± 11 *	165 ± 11 *	162 ± 12 *	163 ± 12 +	*F* = 0.196 *p* = 0.899	***F* = 5.678** ***p* = 0.026**	*F* = 1.022 *p* = 0.388
% of HR_max_ (%)	81 ± 7	81 ± 6	82 ± 5	82 ± 4	86 ± 4	85 ± 4	84 ± 5	84 ± 4	*F* = 0.078 *p* = 0.972	*F* = 3.705 *p* = 0.067	*F* = 1.490 *p* = 0.225
Blood lactate (mmol⋅L^–1^)	1.96 ± 0.64	2.03 ± 0.62	1.92 ± 0.75	1.80 ± 0.56	1.54 ± 0.35	1.82 ± 0.46	1.65 ± 0.35	1.73 ± 0.40	*F* = 1.850 *p* = 0.528	*F* = 1.072 *p* = 0.312	*F* = 0.528 *p* = 0.664
$\dot{\text{V}}$O_2_ (ml⋅kg^–1^⋅min^–1^)	31.6 ± 6.8	32.8 ± 6.9	32.4 ± 6.9	33.0 ± 4.7	34.1 ± 3.8	34.4 ± 5.7	34.1 ± 4.5	33.2 ± 4.6	*F* = 1.473 *p* = 0.246	*F* = 0.020 *p* = 0.889	*F* = 0.307 *p* = 0.717
**Anaerobic threshold**											
Heart rate (bpm)	175 ± 8 *	176 ± 8 *	176 ± 8 +	178 ± 8	184 ± 8 *	183 ± 9 *	180 ± 10 +	183 ± 9	*F* = 1.538 *p* = 0.213	***F* = 5.391** ***p* = 0.030**	*F* = 1.827 *p* = 0.151
Percentage of HR_max_ (%)	92 ± 3 *	92 ± 2 *	92 ± 2 *	93 ± 2 +	95 ± 2 *	94 ± 2 *	95 ± 2 *	94 ± 2 +	*F* = 0.773 *p* = 0.513	***F* = 6.629** ***p* = 0.017**	***F* = 3.437** ***p* = 0.022**
Blood lactate (mmol⋅L^–1^)	4.02 ± 0.85	4.09 ± 1.00	3.78 ± 0.4	4.01 ± 0.87	3.89 ± 0.83	3.52 ± 0.83	3.49 ± 0.83	3.82 ± 0.55	*F* = 1.850 *p* = 0.147	*F* = 1.072 *p* = 0.312	*F* = 0.528 *p* = 0.664
$\dot{\text{V}}$O_2_ (ml⋅kg^–1^⋅min^–1^)	39.1 ± 5.4	39.7 ± 6.8	39.8 ± 5.5	40.7 ± 4.2	40.5 ± 4.3	41.2 ± 4.9	40.4 ± 4.2	39.0 ± 4.5	*F* = 0.133 *p* = 0.854	*F* = 0.328 *p* = 0.575	*F* = 1.198 *p* = 0.312
**Maximal effort**											
Heart rate (bpm)	190 ± 8	192 ± 8	191 ± 8	191 ± 9	195 ± 10	194 ± 9	192 ± 10	193 ± 13	*F* = 1.661 *p* = 0.184	*F* = 1.855 *p* = 0.187	*F* = 2.763 *p* = 0.065
Blood lactate (mmol⋅L^–1^)	11.2 ± 3.4	11.4 ± 3.3	11.3 ± 3.1	10.4 ± 2.2	9.6 ± 2.3	10.2 ± 2.7	8.9 ± 2.7	9.7 ± 2.9	***F* = 2.828** ***p* = 0.045**	*F* = 0.879 *p* = 0.359	*F* = 2.456 *p* = 0.071
$\dot{\text{V}}$O_2peak_ (ml⋅kg^–1^⋅min^–1^)	44.1 ± 5.8	46.4 ± 6.4	46.7 ± 5.3	46.1 ± 5.4	44.1 ± 4.8	47.2 ± 4.4	45.5 ± 3.6	44.9 ± 4.5	*F* = 0.552 *p* = 0.649	*F* = 0.849 *p* = 0.368	*F* = 0.249 *p* = 0.862
$\dot{\text{V}}$O_2peak_ (L⋅min^–1^)	2.97 ± 0.37	3.00 ± 0.40	3.00 ± 0.45	3.02 ± 0.35	2.81 ± 0.38	2.84 ± 0.32	2.77 ± 0.30	2.80 ± 0.34	*F* = 1.933 *p* = 0.135	*F* = 1.799 *p* = 0.197	*F* = 0.314 *p* = 0.815
RUN_peak_ (km⋅h^–1^)	14.8 ± 1.4	15.1 ± 1.5	15.1 ± 1.4	14.9 ± 1.4	14.5 ± 1.3	14.9 ± 1.4	14.9 ± 1.3	14.8 ± 1.6	*F* = 0.675 *p* = 0.570	*F* = 0.737 *p* = 0.400	*F* = 1.910 *p* = 0.136
RUN_total_ (min:s)	27:50 ± 4:31	29:19 ± 4:29	29:06 ± 4:11	28:25 ± 4:02	27:18 ± 3:14	27:31 ± 4:00	27:26 ± 3:58	27:55 ± 4:23	*F* = 0.479 *p* = 0.698	*F* = 1.257 *p* = 0.274	*F* = 1.526 *p* = 0.216

Values are presented as mean ± SD and significant findings are denoted in bold. Simple main effects for group at each phase are denoted by “*” in the table (p < 0.05) and trends are indicated by “+”.

### Aerobic Threshold

The Bla, $\dot{\text{V}}$O_2_ and HR relative to HR_max_ associated with AerT remained stable over phases in EUM and CHC and no group differences were observed between these variables. A main effect for group was observed in HR at AerT (*p* = 0.026, partial η^2^ = 0.205) indicating that when HR at AerT across phase for each group is considered there may be significant difference between groups over phase. Analysis of simple main effects indicate a tendency for HR at AerT means to be lower in EUM than CHC (bleeding, *p* = 0.030, *g* = 0.94, *medium*; mid-FOL/active 1, *p* = 0.020, *g* = 0.82, *medium*; ovulation/active 2, *p* = 0.044, *g* = 0.5, *medium*; and mid LUT/inactive, *p* = 0.051, *g* = 0.61, *medium*).

### Anaerobic Threshold

The Bla and $\dot{\text{V}}$O_2_ associated with AnaT remained stable over phases in EUM and CHC and no differences between groups were observed. A main effect for group was observed in HR at AnaT (*p* = 0.030, partial η^2^ = 0.197) indicating that when HR at AnaT across phase for each group is considered there may be significant difference between groups over phase. Analysis of simple main effects indicate a tendency for HR at AnaT means to be lower in EUM than CHC (bleeding, *p* = 0.015, *g* = 1.13, *large*; mid-FOL/active 1, *p* = 0.016, *g* = 0.83, *medium*; ovulation/active 2, *p* = 0.067, *g* = 0.45, *small*; and mid LUT/inactive, *p* = 0.094, *g* = 0.59, *medium*).

When HR was analyzed relative to HR_max_ at AnaT a significant phase x group interaction was observed (*p* = 0.022, partial η^2^ = 0.135). This interaction was determined to reside between bleeding and mid LUT/inactive (*p* = 0.14, partial η^2^ = 0.237). In addition, a significant main effect for group (*p* = 0.017, partial η^2^ = 0.232) indicating that when HR was analyzed relative to HR_max_ at AnaT across phase for each group is considered there may be significant difference between groups over phase. Further analysis revealed a tendency for HR relative to HR_max_ at AnaT to be lower in EUM than CHC (bleeding, *p* = 0.031, *g* = 1.15, *large*; mid-FOL/active 1, *p* = 0.005, *g* = 1.00, *large*; ovulation/active 2, *p* = 0.010, *g* = 1.00, *large*, mid LUT/inactive, *p* = 0.800, *g* = 0.50, *medium*). The main effect for group observed in HR relative to HR_max_ at AnaT appears to be meaningful even in the presence of the significant interaction due to the observation that CHC generally has higher means of HR relative to HR_max_ at AnaT than EUM.

### Maximal Effort

At maximal effort (just prior to volitional exhaustion), a main effect for phase (*p* = 0.045, partial η^2^ = 0.114) was observed in Bla indicating that when both groups are considered there may be significant fluctuation in Bla over phase. Simple main effects for EUM (*p* = 0.020) and CHC (*p* = 0.120) indicate that this main effect could be driven by significant fluctuation in Bla in EUM, however, further evaluation of EUM over phase revealed no statistically significant differences between phases in Bla.

**FIGURE 2:**
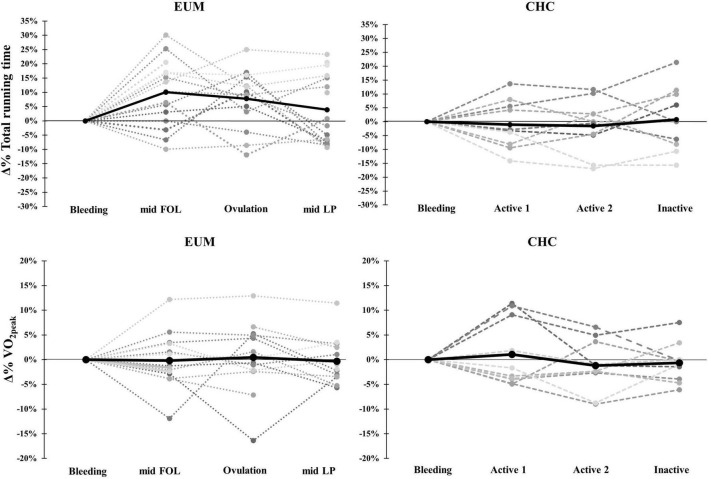
Individual Δ% changes in total running time and $\dot{\text{V}}$O_2peak_ in eumenorrheic (EUM) participants and monophasic hormonal contraceptive users (CHC). The solid black line represents the group mean.

Heart rate values measured at maximal effort were unchanged across phases in EUM and CHC and no between-group differences were observed. Both absolute and relative $\dot{\text{V}}$O_2peak_ remained unchanged across phases in EUM and CHC while no difference between groups was observed. No statistically significant differences between phases or groups were observed for RUN_peak_, or RUN_total_ (Table 4). Figure 2 shows individual percent change from bleeding for RUN_total_ and relative $\dot{\text{V}}$O_2peak_.

## Discussion

In the present study, incremental treadmill testing was performed at four hormonally unique time-points over a MC, and comparable time points during a HC-cycle, in order to elucidate the possible influence of reproductive hormones on physiological variables typically measured and monitored during incremental treadmill testing. Our data suggest that MC and HC phases do not systematically influence physiological variables monitored during incremental treadmill testing. Likewise, testing outcomes are not influenced by MC or HC phase although recording MC phase or HC use may be useful for interpretation of results at an individual level when performing incremental aerobic testing. In the present study a tendency for HR to be lower in EUM than CHC users at AerT and AnaT was observed.

### Menstrual Cycle

The present results suggest that endogenous reproductive hormonal fluctuation characteristic of the MC in eumenorrheic women does not cause significant changes in body mass, maximal performance (RUN_peak_ or RUN_total_) or physiological responses (HR, Bla, or $\dot{\text{V}}$O_2_) at AerT, AnaT, and maximal effort. With regard to body mass fluctuations in relation to endogenous hormonal profiles, research is equivocal with reports suggesting both significant fluctuation (Tomazo-Ravnik and Jakopiè, 2006) as well as no changes.

The lack of significant MC phase-based fluctuation in absolute HR are in line with Jurkowski et al. (1981), who reported similar responses of HR, cardiac output, and stroke volume during both incremental and steady-state cycling exercise in the mid FOL and mid LUT phases, concluding that central cardiovascular response to exercise is not influenced by MC phase. Similarly, Freemas et al. (2021) did not observe any differences in HR between mid FOL and mid LUT during an 8-km cycling time-trial. These findings are, however, not consistent with Rael et al. (2021) who reported lower HR early in FOL compared to late FOL (coinciding with the LH surge indicating ovulation) during high-intensity interval training (8 × 3-min bouts at 85% of maximal aerobic speed with 90-s recovery at 30% of maximal aerobic speed) in endurance-trained women with eumenorrheic cycles. Likewise, a lower HR was observed in FOL compared to LUT during 40 min of running at 75% of individual maximal aerobic speed by Barba-Moreno et al. (2019), an observation attributed to the higher core temperature associated with LUT that is suggested to increase cardiovascular strain (Lebrun, 1993).

The stability of absolute and relative $\dot{\text{V}}$O_2peak_ (Dean et al., 2003; Smith et al., 2015) and submaximal $\dot{\text{V}}$O_2_ observed at AerT and AnaT over the MC appear to be consistent with previous research. Plasma volume and pulmonary diffusion capacity are reported to increase during exercise when E2 concentrations increase, such as in LUT, however, this does not appear to affect $\dot{\text{V}}$O_2peak_ (Smith et al., 2015). Freemas et al. (2021) did not observe differences in absolute $\dot{\text{V}}$O_2_, $\dot{\text{V}}$CO_2_, or VE during an 8-km cycling time-trial between mid FOL and mid LUT. In contrast, Barba-Moreno et al. (2019) observed a higher submaximal $\dot{\text{V}}$O_2_ at mid FOL versus early FOL during running at 75% of maximal aerobic running speed in endurance trained women. Similarly, they reported that tidal volume and ventilatory equivalents of O_2_ and CO_2_ during LUT were higher during mid FOL versus early FOL, suggesting that cardiorespiratory efficiency may be lower during mid FOL (Barba-Moreno et al., 2019). The differences between these findings may be related to methodological approaches as physiological demands, e.g., 8 km cycling time-trial testing at mid FOL and mid LUT in Freemas et al. (2021); continuous submaximal running at early and mid FOL as well as during LUT in Barba-Moreno et al. (2019); and incremental treadmill running to volitional exhaustion in the present study differ. Furthermore, the timing of measurements within MC phases is not consistent between studies.

The stability in Bla concentrations at AerT and AnaT over the MC observed in the present study are in agreement with Mattu et al. (2020), who reported no differences in Bla after maximal exercise or 30-min constant-load cycling trials between mid FOL and mid LUT. Likewise, no changes in lactate threshold (comparable to AnaT) were observed between early FOL, mid FOL, and mid LUT phases of the MC during a graded, maximal exercise test (Dean et al., 2003) and no differences in Bla were observed between mid FOL and late LUT in eumenorrheic participants during 20-s of anaerobic exercise followed by 100-s of aerobic exercise (Lynch and Nimmo, 1998). Research indicates that fluctuations in E2 and P4 might influence metabolic substrate use including fat oxidation (Jurkowski et al., 1981; Hackney et al., 2007) where lipid metabolism during LUT may be enhanced, particularly at lower intensities (Hackney et al., 1994). This observation, generally reflected in lower RER and Bla values suggests a glycogen sparing effect (Hackney, 1999) that may be advantageous for endurance type exercise (Nicklas et al., 1989). Feeding may be a factor mediating the differences observed in substrate metabolism over phase, as carbohydrate supplementation is reported to mitigate differences observed between LUT and FOL in rates of glucose appearance and disappearance as well as total contribution of carbohydrate to energy expenditure (Campbell et al., 2001). It is possible that the sports bar consumed prior to aerobic testing (see section “Materials And Methods”) influenced Bla in the present study, although our approach was standardized for each participant, and practically speaking, it is unlikely for an athlete to perform such incremental treadmill testing in a fasted state.

### Hormonal Contraceptive Cycle

The present results suggest that exogenous reproductive hormone use (monophasic combined HC use) and the resulting endogenous hormonal milieu does not cause significant changes in body mass, maximal performance (RUN_peak_, RUN_total_) or physiological responses (HR, Bla, or $\dot{\text{V}}$O_2_) at AerT, AnaT, or maximal effort, across the HC cycle. Monophasic HCs, such as those used by participants in the present study (see Table 2), provide a relatively stable hormonal condition for 21–24 active days followed by 4–7 hormone-free (inactive) days (Schlaff et al., 2004), although endogenous hormonal profiles may vary considerably between individuals even when HCs employ the same mechanism of action (Elliott-Sale et al., 2013).

The relative stability of submaximal (AerT and AnaT) HR across the HC-cycle is consistent with the findings of Barba-Moreno et al. (2019) and Mattu et al. (2020), who examined the difference between active and inactive phases of HC use by means of a steady-state endurance and a maximal lactate steady-state and ramp-incremental tests, respectively. The lack of significant differences in Bla at AerT and AnaT across the HC-cycle are also in agreement with Mattu et al. (2020), who observed no differences in Bla after maximal exercise between inactive and active pill phases. In contrast, Lynch and Nimmo (1998) observed a higher peak Bla within one week of taking HC compared to a test performed one week later in HC users performing intermittent exercise [20-s of anaerobic exercise followed by 100-s of aerobic exercise (Lynch and Nimmo, 1998)].

The lack of differences in submaximal HR and $\dot{\text{V}}$O_2_ between active and inactive HC phases are in agreement with those of Rechichi et al. (2009), in which HC phase was not observed to have a systematic effect on 1-h cycling performance in female athletes, although cyclic variation in other ventilatory variables was noted (Rechichi et al., 2009). Likewise, Vaiksaar et al. (2011) found no differences between “phases” (HC-cycle day 8 ± 3 versus day 20 ± 2) in terms of power output, HR, $\dot{\text{V}}$O_2_, CO_2_ production, VE, mean RER or ventilatory equivalents of O_2_ and CO_2_ in rowers who were using a monophasic HC (Vaiksaar et al., 2011). In contrast, significant increases in VE, breathing frequency, and ventilatory equivalents for O_2_ and CO_2_ were observed in the active (hormonal) phase versus the inactive phase by Barba-Moreno et al. (2019), who suggested slightly decreased cardiorespiratory efficiency during active HC phases versus the inactive phase.

### Group Differences

The primary purpose of the present study was not to compare groups, however, it is worth noting that in this relatively homogeneous population HR at AerT and AnaT (i.e., submaximal intensities) tended to be higher in CHC than EUM, a difference that was accompanied by medium to large effect sizes. These differences were also present when AnaT was analyzed relative to HR_max_, where effect sizes were medium or large. This latter finding should be interpreted with caution as HR relative to HR_max_ is not based off a fixed HR_max_, but testing day HR_max_, which shows great SDs at maxial effort. The difference in absolute HR observed between CHC and EUM may be explained by E2. Increased concentrations of E2 are known to increase vasodilation and decrease vascular resistance, resulting in decreased HR and blood pressure where E2 concentrations are generally higher in EUM than in CHC. No difference in HR is observed at maximal effort, presumably because HR_max_ is determined to a large extent by age rather than sex (endogenous hormone concentrations) or even training status (Tanaka et al., 2001). These findings emphasize the necessity to prescribe HR-based training individually.

### Strengths and Weaknesses

The present study included several strengths and weaknesses. Our participants were relatively homogeneous in terms of age while all participants were physically active rather than sedentary. Our inclusion of both EUM and CHC in this investigation provides additional perspectives to scientists and practitioners while our findings illustrate potential differences between EUM and CHC in terms of hormonal profiles over the MC and HC-cycles while demonstrating how endocrine fluctuations might influence variables used to monitor and assess aerobic fitness. The study design was rigorous and included four time-points rather than the usual two or three used in many studies. Furthermore, in order to mitigate the influence of the learning effect, the order of starting the tests was randomized, although this did mean that parts of two consecutive MCs or HCs may have been used rather than a single MC or HC-cycle. Nevertheless, we incorporated both prospective determination of MC phases as well as retrospective confirmation of both MC and HC phases according to current recommendations for best practice (Elliott-Sale et al., 2020b). Furthermore, we collected nutritional data and strived to perform testing in a standardized fed state as well as at the same time of day (Ihalainen et al., 2021). We must acknowledge that different HC formulations and dosages individually affect endogenous hormonal profiles (Elliott-Sale et al., 2013), while also recognizing that eumenorrheic cycles display variation (MacNutt et al., 2012). More frequent hormonal sampling and the use of different kinds of aerobic performance testing could further help to elucidate the possible influence of endogenous hormonal profile on physiological variables monitored in testing. We also acknowledge that a plethora of other endogenous and exogenous factors including, but not limited to neuromuscular performance, nutrition, sleep, and motivation, may have a greater influence on physiological variables monitored during testing as well as test outcomes than MC or HC phase alone.

## Conclusion

This study provides evidence that endogenous or exogenous-induced hormonal fluctuations over MC and HC-cycles do not systematically influence physiological variables used to assess aerobic fitness, and do not significantly affect the interpretation of incremental treadmill running tests of young, healthy, physically active women. The observed differences in HR at AerT and AnaT observed between EUM and CHC should be noted and used as a reminder that HR-based training be prescribed individually. Athletes, coaches, and researchers are encouraged to record MC or HC-cycle phase when completing testing and to consider MC phase particularly when analyzing or comparing physiological responses at AerT and AnaT (e.g., Bla) over a series of tests as individual differences in performance over phase are possible and subjective feelings related to MC or HC phase may be of importance. Further investigation of individual hormonal profiles and their effects on individual variables related to performance may be warranted.

## Data Availability Statement

The raw data supporting the conclusions of this article will be made available by the authors, without undue reservation.

## Ethics Statement

The studies involving human participants were reviewed and approved by the Ethics Committee of the University of Jyväskylä, Jyväskylä, Finland. The patients/participants provided their written informed consent to participate in this study.

## Author Contributions

JI, RT-M, and AH contributed to conception and design of the study. RT-M and AR performed the statistical analysis. RT-M, AR, HP, and JI wrote the first draft of the manuscript. All authors contributed to manuscript revision, read, and approved the submitted version.

## Conflict of Interest

The authors declare that the research was conducted in the absence of any commercial or financial relationships that could be construed as a potential conflict of interest.

## Publisher’s Note

All claims expressed in this article are solely those of the authors and do not necessarily represent those of their affiliated organizations, or those of the publisher, the editors and the reviewers. Any product that may be evaluated in this article, or claim that may be made by its manufacturer, is not guaranteed or endorsed by the publisher.
